# 3-Benzoyl-1,1-dibenzyl­thio­urea

**DOI:** 10.1107/S1600536810036226

**Published:** 2010-09-18

**Authors:** N. Gunasekaran, R. Karvembu, Seik Weng Ng, Edward R. T. Tiekink

**Affiliations:** a Department of Chemistry, National Institute of Technology, Tiruchirappalli 620 015, India; b Department of Chemistry, University of Malaya, 50603 Kuala Lumpur, Malaysia

## Abstract

Two independent thio­urea mol­ecules comprise the asymmetric unit of the title compound, C_22_H_20_N_2_OS. The central N–C(=S)N(H)C(=O) atoms in each mol­ecule are virtually superimposable and each is twisted [C—N—C—S torsion angles = 121.3 (3) and −62.3 (4)°]. The mol­ecules differ only in terms of the relative orientations of the benzyl benzene rings [major difference between the C—N—C—C torsion angles of −146.6 (3) and −132.9 (3)°]. The presence of N—H⋯S hydrogen bonding leads to the formation of supra­molecular chains along the *a* axis. These are consolidated in the crystal packing by C—H⋯O inter­actions. The crystal was found to be a combined non-merohedral and racemic twin (twin law 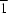
00/0
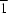
0/001), with the fractional contribution of the minor components being approximately 9 and 28%.

## Related literature

For our studies of thio­urea and its derivatives, see: Gunasekaran *et al.* (2010 ▶). For the biological activity of thio­urea derivatives, see: Venkatachalam *et al.* (2004 ▶); Yuan *et al.* (2001 ▶); Zhou *et al.* (2004 ▶). For additional geometric analysis, see: Spek (2009 ▶).
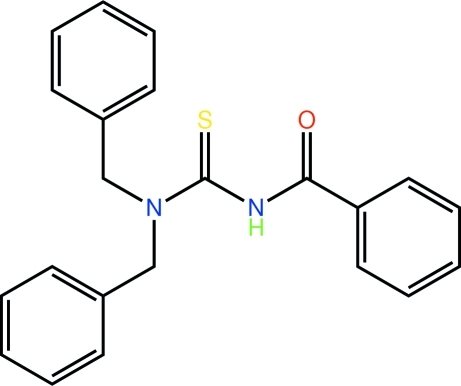


## Experimental

### 

#### Crystal data

C_22_H_20_N_2_OS
*M* *_r_* = 360.46
Monoclinic, 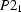

*a* = 7.7338 (5) Å
*b* = 24.3478 (16) Å
*c* = 9.8593 (6) Å
β = 90.074 (1)°
*V* = 1856.5 (2) Å^3^
*Z* = 4
Mo *K*α radiation
μ = 0.19 mm^−1^
*T* = 100 K
0.30 × 0.20 × 0.10 mm

#### Data collection

Bruker SMART APEX diffractometer
Absorption correction: multi-scan (*SADABS*; Sheldrick, 1996 ▶) *T*_min_ = 0.946, *T*_max_ = 0.982
17536 measured reflections
8469 independent reflections
7807 reflections with *I* > 2σ(*I*)
*R*_int_ = 0.055

#### Refinement

*R*[*F* ^2^ > 2σ(*F* ^2^)] = 0.058
*wR*(*F* ^2^) = 0.157
*S* = 1.03
8469 reflections
470 parameters
1 restraint
H-atom parameters constrained
Δρ_max_ = 1.27 e Å^−3^
Δρ_min_ = −0.36 e Å^−3^
Absolute structure: Flack (1983 ▶), 4101 Friedel pairs
Flack parameter: 0.25 (8)

### 

Data collection: *APEX2* (Bruker, 2008 ▶); cell refinement: *SAINT* (Bruker, 2008 ▶); data reduction: *SAINT*; program(s) used to solve structure: *SHELXS97* (Sheldrick, 2008 ▶); program(s) used to refine structure: *SHELXL97* (Sheldrick, 2008 ▶); molecular graphics: *ORTEP-3* (Farrugia, 1997 ▶), *DIAMOND* (Brandenburg, 2006 ▶) and *Qmol* (Gans & Shalloway, 2001 ▶); software used to prepare material for publication: *publCIF* (Westrip, 2010 ▶).

## Supplementary Material

Crystal structure: contains datablocks global, I. DOI: 10.1107/S1600536810036226/lh5120sup1.cif

Structure factors: contains datablocks I. DOI: 10.1107/S1600536810036226/lh5120Isup2.hkl

Additional supplementary materials:  crystallographic information; 3D view; checkCIF report

## Figures and Tables

**Table 1:** Hydrogen-bond geometry (Å, °)

*D*—H⋯*A*	*D*—H	H⋯*A*	*D*⋯*A*	*D*—H⋯*A*
N2—H2⋯S2	0.86	2.54	3.334 (3)	154
N4—H4⋯S1^i^	0.86	2.54	3.334 (3)	154
C13—H13⋯O2^ii^	0.95	2.57	3.193 (5)	124
C14—H14⋯O2^ii^	0.95	2.60	3.207 (5)	122
C25—H25⋯O1^iii^	0.95	2.55	3.228 (4)	129

Symmetry codes: (i) 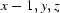; (ii) 
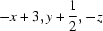
; (iii) 
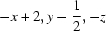
.

**Fig. 1.**
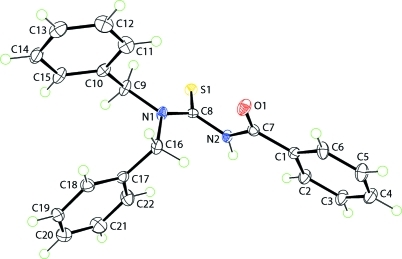
The molecular structure of the first independent molecule in (I) showing the atom-labelling scheme and displacement ellipsoids at the 50% probability level.

**Fig. 2.**
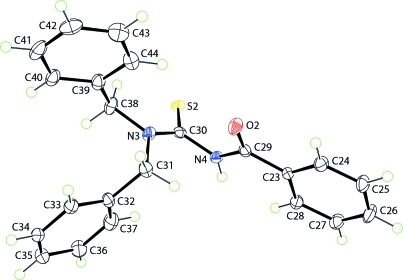
The molecular structure of the second independent molecule in (I) showing the atom-labelling scheme and displacement ellipsoids at the 50% probability level.

**Fig. 3.**
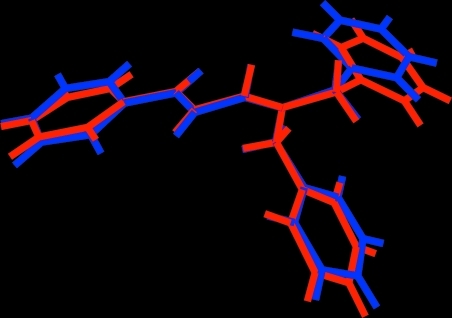
Overlay diagram of the first independent molecule (shown in red) and the second independent molecule (shown in blue).

**Fig. 4.**
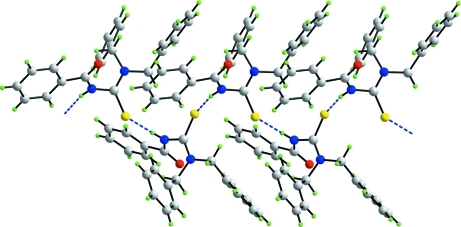
Linear supramolecular chain along the a axis in (I) mediated by N–H···S hydrogen bonding, shown as blue dashed lines.

**Fig. 5.**
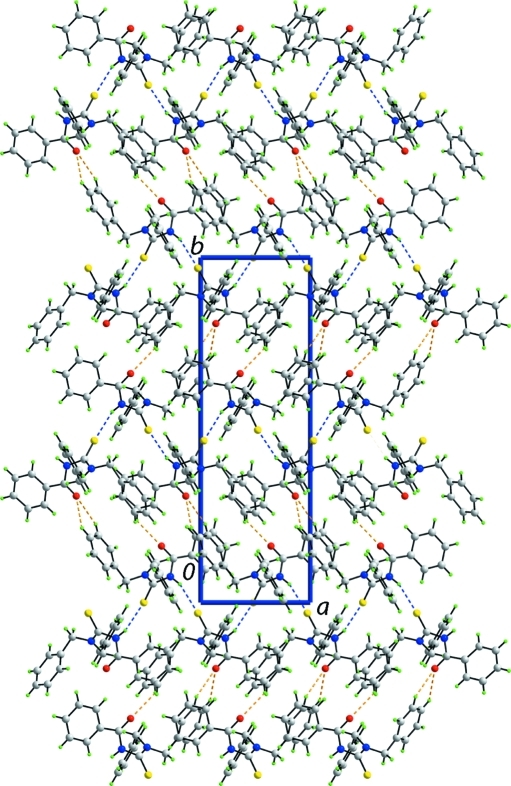
Unit-cell contents shown in projection down the c axis in (I). The N–H···S hydrogen bonding and C–H···O contacts are shown as blue and orange dashed lines, respectively.

## supplementary crystallographic information

### Comment

The title compound, (I), was investigated in continuation of studies (Gunasekaran *et al.*, 2010) of thiourea and its derivatives, which are useful as anti-tumour, anti-fungal, anti-bacterial, insecticidal, herbicidal, pesticidal agents, and plant-growth regulators (Venkatachalam *et al.*, 2004; Yuan *et al.*, 2001; Zhou *et al.*, 2004).

Two independent molecules comprise the asymmetric unit of (I). The central N–C(═S)N(H)C(═O) atoms of the first independent molecule, Fig. 1, are virtually super-imposable upon those of the second, Fig. 2. The C7–N2–C8–S1 and C29–N4 –C30–N3 torsion angles of 121.3 (3) and -62.3 (4) °, respectively, indicate significant twists in the central part of each molecule. The major differences between the molecules relate to the orientations of the benzene rings as indicated in Fig. 3. The major conformational difference is quantified in the C8–N1–C9–C10 and C30–N3–C38–C39 torsion angles -146.6 (3) and -132.9 (3) °, respectively. The r.m.s. deviations for bond distances and angles are 0.0105 Å and 0.651 °, respectively (Spek, 2009).

The most notable feature in the crystal packing is the formation of supramolecular chains along the *a* axis mediated by N–H···S hydrogen bonding, Fig. 4 and Table 1. The chains pack in the *ac* plane and stack along the *b* axis with the primary connections along this axis being of the type C–H···O, Fig. 5 and Table 1.

### Experimental

A solution of benzoyl chloride (0.7029 g, 5 mmol) in acetone (50 ml) was added drop wise to a suspension of potassium thiocyanate (0.4859 g, 5 mmol) in anhydrous acetone (50 ml). The reaction mixture was heated under reflux for 45 minutes and then cooled to room temperature. A solution of dibenzyl amine (0.9864 g, 5 mmol) in acetone (30 ml) was added and the resulting mixture was stirred for 2 h. Hydrochloric acid (0.1 N, 300 ml) was added and the resulting white solid was filtered, washed with water and dried *in vacuo.* Single crystals were grown at room temperature from its ethyl acetate solution by the diffusion of diethyl ether vapour. Yield 78%; *M*. Pt. 403 K; FT—IR (KBr) ν(N–H) 3239, ν(C═O) 1690, ν(C═S) 1314 cm^-1^.

### Refinement

Carbon-bound H-atoms were placed in calculated positions (N–H = 0.86 Å and C–H = 0.95 to 0.99 Å) and were included in the refinement in the riding model approximation, with *U*_iso_(H) set to 1.2*U*_equiv_(N, C). The maximum and minimum residual electron density peaks of 1.27 and 0.36 e Å^-3^, respectively, were located 1.65 Å and 0.89 Å from the H38*a* and S2 atoms, respectively. As the structure is a non-merohedral twin that belongs to a non-centric space group, the non-merohedral twinning and racemic twinning were treated in combination. The twin law -1 0 0 / 0 - 1 0 / 0 0 1 was used as the monoclinic unit cell emulated an orthorhombic unit cell. The Flack parameter refined to 0.25 (8).

### Crystal data

**Table:**

C_22_H_20_N_2_OS	*F*(000) = 760
*M**_r_* = 360.46	*D*_x_ = 1.290 Mg m^−^^3^
Monoclinic, *P*2_1_	Mo *K*α radiation, λ = 0.71073 Å
Hall symbol: P 2yb	Cell parameters from 6510 reflections
*a* = 7.7338 (5) Å	θ = 2.2–28.1°
*b* = 24.3478 (16) Å	µ = 0.19 mm^−^^1^
*c* = 9.8593 (6) Å	*T* = 100 K
β = 90.074 (1)°	Block, colourless
*V* = 1856.5 (2) Å^3^	0.30 × 0.20 × 0.10 mm
*Z* = 4	

### Data collection

**Table:**

Bruker SMART APEX diffractometer	8469 independent reflections
Radiation source: fine-focus sealed tube	7807 reflections with *I* > 2σ(*I*)
graphite	*R*_int_ = 0.055
ω scans	θ_max_ = 27.5°, θ_min_ = 0.8°
Absorption correction: multi-scan (*SADABS*; Sheldrick, 1996)	*h* = −10→10
*T*_min_ = 0.946, *T*_max_ = 0.982	*k* = −31→31
17536 measured reflections	*l* = −12→11

### Refinement

**Table:**

Refinement on *F*^2^	Secondary atom site location: difference Fourier map
Least-squares matrix: full	Hydrogen site location: inferred from neighbouring sites
*R*[*F*^2^ > 2σ(*F*^2^)] = 0.058	H-atom parameters constrained
*wR*(*F*^2^) = 0.157	*w* = 1/[σ^2^(*F*_o_^2^) + (0.0934*P*)^2^ + 0.2767*P*] where *P* = (*F*_o_^2^ + 2*F*_c_^2^)/3
*S* = 1.03	(Δ/σ)_max_ < 0.001
8469 reflections	Δρ_max_ = 1.27 e Å^−^^3^
470 parameters	Δρ_min_ = −0.36 e Å^−^^3^
1 restraint	Absolute structure: Flack (1983), 4101 Friedel pairs
Primary atom site location: structure-invariant direct methods	Flack parameter: 0.25 (8)

### Fractional atomic coordinates and isotropic or equivalent isotropic displacement parameters (Å^2^)

**Table:**

	*x*	*y*	*z*	*U*_iso_*/*U*_eq_	
S1	1.52015 (10)	0.49995 (3)	0.21954 (8)	0.01637 (17)	
S2	1.02727 (11)	0.46914 (3)	0.10081 (9)	0.01665 (17)	
O1	1.3589 (3)	0.66362 (10)	0.2194 (3)	0.0228 (6)	
O2	0.8629 (3)	0.30856 (10)	0.0894 (3)	0.0239 (6)	
N1	1.5083 (4)	0.58006 (11)	0.0339 (3)	0.0157 (6)	
N2	1.2782 (4)	0.57501 (10)	0.1861 (3)	0.0140 (5)	
H2	1.1911	0.5531	0.1843	0.017*	
N3	1.0001 (4)	0.38816 (12)	0.2845 (3)	0.0179 (6)	
N4	0.7766 (4)	0.39659 (11)	0.1265 (3)	0.0162 (6)	
H4	0.6899	0.4187	0.1277	0.019*	
C1	1.0820 (4)	0.63713 (13)	0.3027 (3)	0.0154 (6)	
C2	0.9587 (5)	0.59635 (14)	0.3240 (4)	0.0204 (7)	
H2A	0.9822	0.5597	0.2969	0.024*	
C3	0.8020 (5)	0.60887 (16)	0.3846 (4)	0.0242 (8)	
H3	0.7183	0.5809	0.3986	0.029*	
C4	0.7672 (5)	0.66233 (15)	0.4247 (4)	0.0239 (8)	
H4A	0.6601	0.6708	0.4668	0.029*	
C5	0.8880 (5)	0.70339 (15)	0.4038 (4)	0.0240 (8)	
H5	0.8635	0.7400	0.4310	0.029*	
C6	1.0446 (5)	0.69113 (14)	0.3431 (4)	0.0208 (7)	
H6	1.1273	0.7194	0.3287	0.025*	
C7	1.2514 (4)	0.62775 (13)	0.2336 (3)	0.0151 (6)	
C8	1.4372 (4)	0.55502 (13)	0.1409 (3)	0.0139 (6)	
C9	1.6810 (4)	0.56209 (14)	−0.0142 (4)	0.0195 (7)	
H9A	1.6654	0.5336	−0.0849	0.023*	
H9B	1.7441	0.5451	0.0624	0.023*	
C10	1.7895 (4)	0.60781 (13)	−0.0715 (4)	0.0176 (7)	
C11	1.8660 (5)	0.60174 (15)	−0.1997 (4)	0.0214 (7)	
H11	1.8424	0.5700	−0.2527	0.026*	
C12	1.9766 (5)	0.64222 (16)	−0.2494 (4)	0.0234 (8)	
H12	2.0286	0.6380	−0.3360	0.028*	
C13	2.0104 (5)	0.68838 (16)	−0.1727 (4)	0.0252 (8)	
H13	2.0856	0.7159	−0.2069	0.030*	
C14	1.9355 (5)	0.69483 (16)	−0.0462 (4)	0.0272 (8)	
H14	1.9600	0.7267	0.0059	0.033*	
C15	1.8248 (5)	0.65500 (15)	0.0050 (4)	0.0242 (8)	
H15	1.7731	0.6598	0.0916	0.029*	
C16	1.4092 (5)	0.61491 (14)	−0.0616 (3)	0.0178 (7)	
H16A	1.4751	0.6489	−0.0810	0.021*	
H16B	1.2978	0.6255	−0.0197	0.021*	
C17	1.3751 (4)	0.58415 (14)	−0.1933 (3)	0.0163 (6)	
C18	1.4287 (4)	0.60578 (15)	−0.3151 (4)	0.0201 (7)	
H18	1.4929	0.6390	−0.3166	0.024*	
C19	1.3891 (5)	0.57905 (16)	−0.4367 (4)	0.0224 (7)	
H19	1.4247	0.5944	−0.5207	0.027*	
C20	1.2973 (5)	0.52985 (17)	−0.4347 (4)	0.0265 (8)	
H20	1.2704	0.5115	−0.5171	0.032*	
C21	1.2453 (5)	0.50774 (16)	−0.3114 (4)	0.0280 (8)	
H21	1.1833	0.4741	−0.3098	0.034*	
C22	1.2830 (5)	0.53436 (15)	−0.1908 (4)	0.0235 (8)	
H22	1.2468	0.5191	−0.1068	0.028*	
C23	0.5896 (4)	0.33461 (14)	−0.0012 (3)	0.0152 (6)	
C24	0.5720 (5)	0.28392 (14)	−0.0668 (4)	0.0208 (7)	
H24	0.6619	0.2574	−0.0607	0.025*	
C25	0.4246 (5)	0.27233 (15)	−0.1403 (4)	0.0237 (8)	
H25	0.4145	0.2383	−0.1869	0.028*	
C26	0.2900 (5)	0.31064 (17)	−0.1465 (4)	0.0279 (9)	
H26	0.1865	0.3019	−0.1938	0.033*	
C27	0.3071 (5)	0.36096 (15)	−0.0840 (4)	0.0240 (8)	
H27	0.2174	0.3875	−0.0911	0.029*	
C28	0.4566 (4)	0.37286 (14)	−0.0104 (4)	0.0196 (7)	
H28	0.4677	0.4074	0.0337	0.024*	
C29	0.7538 (4)	0.34428 (14)	0.0742 (3)	0.0158 (6)	
C30	0.9368 (4)	0.41485 (13)	0.1778 (3)	0.0153 (6)	
C31	0.8927 (5)	0.35268 (14)	0.3720 (4)	0.0208 (7)	
H31A	0.9526	0.3173	0.3878	0.025*	
H31B	0.7812	0.3449	0.3265	0.025*	
C32	0.8599 (5)	0.38108 (14)	0.5071 (3)	0.0182 (7)	
C33	0.9196 (5)	0.35777 (15)	0.6274 (4)	0.0199 (7)	
H33	0.9831	0.3244	0.6251	0.024*	
C34	0.8866 (5)	0.38311 (16)	0.7499 (4)	0.0221 (7)	
H34	0.9260	0.3668	0.8319	0.026*	
C35	0.7964 (5)	0.43216 (16)	0.7538 (4)	0.0246 (8)	
H35	0.7749	0.4497	0.8382	0.030*	
C36	0.7367 (5)	0.45597 (15)	0.6329 (4)	0.0263 (8)	
H36	0.6738	0.4895	0.6351	0.032*	
C37	0.7702 (5)	0.43033 (16)	0.5101 (4)	0.0271 (8)	
H37	0.7315	0.4466	0.4278	0.033*	
C38	1.1725 (5)	0.40062 (15)	0.3372 (4)	0.0230 (8)	
H38A	1.1607	0.4216	0.4227	0.028*	
H38B	1.2339	0.4242	0.2712	0.028*	
C39	1.2794 (5)	0.35003 (15)	0.3639 (4)	0.0212 (7)	
C40	1.3815 (5)	0.34745 (16)	0.4800 (4)	0.0244 (8)	
H40	1.3763	0.3760	0.5457	0.029*	
C41	1.4926 (5)	0.30219 (17)	0.4997 (5)	0.0304 (9)	
H41	1.5618	0.3001	0.5793	0.036*	
C42	1.5013 (5)	0.26096 (16)	0.4041 (4)	0.0288 (9)	
H42	1.5789	0.2311	0.4167	0.035*	
C43	1.3972 (6)	0.26289 (17)	0.2894 (5)	0.0308 (9)	
H43	1.4020	0.2341	0.2246	0.037*	
C44	1.2852 (5)	0.30727 (16)	0.2692 (4)	0.0255 (8)	
H44	1.2130	0.3084	0.1912	0.031*	

### Atomic displacement parameters (Å^2^)

**Table:**

	*U*^11^	*U*^22^	*U*^33^	*U*^12^	*U*^13^	*U*^23^
S1	0.0164 (4)	0.0154 (4)	0.0173 (4)	0.0015 (3)	−0.0008 (3)	0.0026 (3)
S2	0.0153 (4)	0.0147 (4)	0.0199 (4)	−0.0025 (3)	0.0031 (3)	0.0014 (3)
O1	0.0184 (12)	0.0191 (12)	0.0310 (15)	−0.0043 (10)	0.0077 (11)	−0.0040 (11)
O2	0.0204 (13)	0.0205 (12)	0.0309 (15)	0.0037 (10)	−0.0070 (11)	−0.0070 (11)
N1	0.0158 (14)	0.0158 (13)	0.0154 (14)	0.0053 (11)	0.0039 (11)	0.0037 (10)
N2	0.0145 (13)	0.0118 (12)	0.0157 (14)	−0.0011 (10)	0.0029 (11)	−0.0022 (10)
N3	0.0171 (14)	0.0161 (13)	0.0206 (15)	−0.0071 (11)	−0.0011 (12)	0.0006 (11)
N4	0.0141 (13)	0.0154 (13)	0.0191 (14)	−0.0001 (10)	0.0016 (11)	−0.0022 (11)
C1	0.0141 (15)	0.0159 (15)	0.0162 (16)	0.0013 (12)	−0.0003 (12)	−0.0014 (12)
C2	0.0191 (17)	0.0161 (16)	0.0259 (19)	−0.0012 (13)	0.0042 (14)	−0.0053 (13)
C3	0.0165 (17)	0.0258 (18)	0.030 (2)	−0.0058 (14)	0.0078 (15)	−0.0075 (15)
C4	0.0172 (17)	0.0254 (18)	0.029 (2)	0.0016 (14)	0.0050 (15)	−0.0027 (15)
C5	0.027 (2)	0.0170 (17)	0.027 (2)	0.0042 (14)	0.0069 (16)	−0.0024 (14)
C6	0.0264 (19)	0.0152 (16)	0.0209 (18)	−0.0008 (13)	0.0076 (14)	−0.0031 (13)
C7	0.0164 (16)	0.0163 (15)	0.0125 (15)	0.0004 (12)	−0.0008 (12)	0.0005 (12)
C8	0.0109 (14)	0.0167 (15)	0.0141 (15)	−0.0023 (12)	−0.0017 (12)	−0.0025 (12)
C9	0.0160 (16)	0.0184 (16)	0.0242 (18)	0.0031 (13)	0.0087 (14)	0.0039 (14)
C10	0.0139 (15)	0.0160 (15)	0.0228 (18)	−0.0004 (12)	0.0015 (13)	0.0021 (13)
C11	0.0162 (16)	0.0210 (17)	0.0271 (19)	−0.0009 (13)	0.0072 (14)	0.0023 (14)
C12	0.0160 (16)	0.0312 (19)	0.0231 (19)	0.0009 (14)	0.0057 (14)	0.0039 (15)
C13	0.0193 (18)	0.0255 (18)	0.031 (2)	−0.0004 (14)	0.0003 (15)	0.0101 (16)
C14	0.031 (2)	0.0213 (18)	0.029 (2)	−0.0020 (15)	−0.0030 (16)	0.0002 (15)
C15	0.030 (2)	0.0186 (17)	0.0242 (19)	−0.0007 (14)	0.0005 (15)	0.0011 (14)
C16	0.0190 (16)	0.0179 (15)	0.0163 (16)	0.0058 (12)	0.0048 (13)	0.0052 (13)
C17	0.0130 (15)	0.0192 (15)	0.0166 (16)	0.0035 (12)	0.0004 (12)	0.0034 (13)
C18	0.0195 (17)	0.0227 (17)	0.0180 (17)	−0.0022 (13)	−0.0012 (13)	0.0027 (14)
C19	0.0211 (18)	0.032 (2)	0.0141 (17)	−0.0012 (15)	0.0003 (14)	0.0044 (14)
C20	0.0238 (19)	0.031 (2)	0.0248 (19)	−0.0036 (15)	−0.0021 (15)	−0.0018 (16)
C21	0.031 (2)	0.0244 (19)	0.029 (2)	−0.0068 (15)	−0.0015 (16)	0.0006 (15)
C22	0.0289 (19)	0.0224 (17)	0.0192 (18)	−0.0051 (15)	0.0052 (15)	0.0039 (14)
C23	0.0145 (15)	0.0179 (15)	0.0132 (15)	−0.0013 (12)	0.0003 (12)	−0.0014 (12)
C24	0.0203 (18)	0.0170 (16)	0.0252 (19)	−0.0012 (13)	−0.0008 (15)	−0.0043 (14)
C25	0.0228 (18)	0.0198 (17)	0.028 (2)	−0.0004 (14)	−0.0053 (15)	−0.0087 (15)
C26	0.0183 (18)	0.0298 (19)	0.036 (2)	−0.0001 (15)	−0.0116 (16)	−0.0074 (17)
C27	0.0180 (18)	0.0263 (19)	0.028 (2)	0.0045 (14)	−0.0039 (15)	−0.0032 (15)
C28	0.0181 (17)	0.0207 (16)	0.0199 (17)	0.0000 (13)	−0.0019 (14)	−0.0019 (13)
C29	0.0175 (16)	0.0178 (15)	0.0121 (15)	0.0004 (12)	0.0002 (12)	0.0000 (12)
C30	0.0155 (15)	0.0130 (14)	0.0172 (16)	−0.0015 (12)	0.0033 (12)	−0.0041 (12)
C31	0.0266 (18)	0.0189 (16)	0.0170 (17)	−0.0093 (14)	0.0004 (14)	0.0010 (13)
C32	0.0219 (17)	0.0185 (16)	0.0142 (16)	−0.0085 (13)	0.0002 (13)	−0.0008 (13)
C33	0.0189 (17)	0.0204 (17)	0.0204 (18)	−0.0023 (13)	−0.0012 (13)	0.0030 (13)
C34	0.0172 (17)	0.0334 (19)	0.0156 (17)	0.0018 (14)	−0.0013 (13)	0.0018 (15)
C35	0.0258 (19)	0.029 (2)	0.0187 (18)	−0.0012 (15)	−0.0002 (15)	−0.0018 (15)
C36	0.027 (2)	0.0238 (19)	0.028 (2)	0.0093 (15)	−0.0025 (16)	−0.0043 (15)
C37	0.027 (2)	0.0275 (19)	0.027 (2)	−0.0015 (15)	−0.0071 (16)	0.0053 (16)
C38	0.0217 (17)	0.0205 (17)	0.027 (2)	−0.0067 (14)	−0.0072 (15)	−0.0003 (14)
C39	0.0210 (18)	0.0235 (17)	0.0190 (17)	−0.0059 (14)	−0.0022 (14)	0.0027 (14)
C40	0.0188 (18)	0.0309 (19)	0.0234 (19)	−0.0093 (15)	−0.0040 (14)	0.0052 (15)
C41	0.0180 (18)	0.036 (2)	0.037 (2)	−0.0102 (16)	−0.0069 (16)	0.0093 (17)
C42	0.0201 (18)	0.0238 (18)	0.043 (2)	−0.0037 (15)	0.0075 (17)	0.0078 (16)
C43	0.034 (2)	0.0236 (19)	0.035 (2)	0.0015 (17)	0.0039 (18)	−0.0001 (16)
C44	0.031 (2)	0.0240 (18)	0.0214 (19)	−0.0031 (15)	−0.0013 (15)	−0.0002 (15)

### Geometric parameters (Å, °)

**Table:**

S1—C8	1.676 (3)	C18—H18	0.9500
S2—C30	1.678 (3)	C19—C20	1.393 (5)
O1—C7	1.214 (4)	C19—H19	0.9500
O2—C29	1.221 (4)	C20—C21	1.390 (6)
N1—C8	1.337 (4)	C20—H20	0.9500
N1—C16	1.480 (4)	C21—C22	1.385 (5)
N1—C9	1.484 (4)	C21—H21	0.9500
N2—C7	1.382 (4)	C22—H22	0.9500
N2—C8	1.396 (4)	C23—C28	1.390 (5)
N2—H2	0.8600	C23—C24	1.400 (5)
N3—C30	1.329 (4)	C23—C29	1.489 (5)
N3—C38	1.462 (4)	C24—C25	1.380 (5)
N3—C31	1.477 (4)	C24—H24	0.9500
N4—C29	1.386 (4)	C25—C26	1.399 (5)
N4—C30	1.410 (4)	C25—H25	0.9500
N4—H4	0.8600	C26—C27	1.378 (5)
C1—C2	1.393 (5)	C26—H26	0.9500
C1—C6	1.404 (5)	C27—C28	1.395 (5)
C1—C7	1.495 (5)	C27—H27	0.9500
C2—C3	1.386 (5)	C28—H28	0.9500
C2—H2A	0.9500	C31—C32	1.522 (5)
C3—C4	1.387 (5)	C31—H31A	0.9900
C3—H3	0.9500	C31—H31B	0.9900
C4—C5	1.384 (5)	C32—C37	1.386 (5)
C4—H4A	0.9500	C32—C33	1.393 (5)
C5—C6	1.384 (5)	C33—C34	1.381 (5)
C5—H5	0.9500	C33—H33	0.9500
C6—H6	0.9500	C34—C35	1.384 (5)
C9—C10	1.504 (5)	C34—H34	0.9500
C9—H9A	0.9900	C35—C36	1.403 (5)
C9—H9B	0.9900	C35—H35	0.9500
C10—C15	1.401 (5)	C36—C37	1.387 (6)
C10—C11	1.405 (5)	C36—H36	0.9500
C11—C12	1.394 (5)	C37—H37	0.9500
C11—H11	0.9500	C38—C39	1.507 (5)
C12—C13	1.379 (6)	C38—H38A	0.9900
C12—H12	0.9500	C38—H38B	0.9900
C13—C14	1.384 (6)	C39—C40	1.391 (5)
C13—H13	0.9500	C39—C44	1.399 (5)
C14—C15	1.389 (5)	C40—C41	1.411 (6)
C14—H14	0.9500	C40—H40	0.9500
C15—H15	0.9500	C41—C42	1.379 (6)
C16—C17	1.522 (5)	C41—H41	0.9500
C16—H16A	0.9900	C42—C43	1.388 (6)
C16—H16B	0.9900	C42—H42	0.9500
C17—C18	1.376 (5)	C43—C44	1.399 (6)
C17—C22	1.406 (5)	C43—H43	0.9500
C18—C19	1.398 (5)	C44—H44	0.9500
			
C8—N1—C16	123.3 (3)	C22—C21—C20	120.6 (3)
C8—N1—C9	119.3 (3)	C22—C21—H21	119.7
C16—N1—C9	115.5 (3)	C20—C21—H21	119.7
C7—N2—C8	124.4 (3)	C21—C22—C17	119.6 (3)
C7—N2—H2	117.8	C21—C22—H22	120.2
C8—N2—H2	117.8	C17—C22—H22	120.2
C30—N3—C38	121.0 (3)	C28—C23—C24	119.3 (3)
C30—N3—C31	122.8 (3)	C28—C23—C29	123.9 (3)
C38—N3—C31	115.3 (3)	C24—C23—C29	116.9 (3)
C29—N4—C30	122.3 (3)	C25—C24—C23	120.2 (3)
C29—N4—H4	118.8	C25—C24—H24	119.9
C30—N4—H4	118.8	C23—C24—H24	119.9
C2—C1—C6	118.9 (3)	C24—C25—C26	120.1 (3)
C2—C1—C7	124.1 (3)	C24—C25—H25	120.0
C6—C1—C7	117.0 (3)	C26—C25—H25	120.0
C3—C2—C1	120.5 (3)	C27—C26—C25	120.2 (3)
C3—C2—H2A	119.8	C27—C26—H26	119.9
C1—C2—H2A	119.8	C25—C26—H26	119.9
C2—C3—C4	119.9 (3)	C26—C27—C28	119.8 (3)
C2—C3—H3	120.0	C26—C27—H27	120.1
C4—C3—H3	120.0	C28—C27—H27	120.1
C5—C4—C3	120.3 (3)	C23—C28—C27	120.5 (3)
C5—C4—H4A	119.9	C23—C28—H28	119.7
C3—C4—H4A	119.9	C27—C28—H28	119.7
C6—C5—C4	120.0 (3)	O2—C29—N4	121.4 (3)
C6—C5—H5	120.0	O2—C29—C23	122.5 (3)
C4—C5—H5	120.0	N4—C29—C23	116.1 (3)
C5—C6—C1	120.3 (3)	N3—C30—N4	116.9 (3)
C5—C6—H6	119.8	N3—C30—S2	126.2 (2)
C1—C6—H6	119.8	N4—C30—S2	116.9 (3)
O1—C7—N2	121.7 (3)	N3—C31—C32	109.9 (3)
O1—C7—C1	122.9 (3)	N3—C31—H31A	109.7
N2—C7—C1	115.4 (3)	C32—C31—H31A	109.7
N1—C8—N2	117.1 (3)	N3—C31—H31B	109.7
N1—C8—S1	124.9 (3)	C32—C31—H31B	109.7
N2—C8—S1	117.9 (2)	H31A—C31—H31B	108.2
N1—C9—C10	113.8 (3)	C37—C32—C33	120.0 (3)
N1—C9—H9A	108.8	C37—C32—C31	119.7 (3)
C10—C9—H9A	108.8	C33—C32—C31	120.3 (3)
N1—C9—H9B	108.8	C34—C33—C32	120.1 (3)
C10—C9—H9B	108.8	C34—C33—H33	119.9
H9A—C9—H9B	107.7	C32—C33—H33	119.9
C15—C10—C11	119.3 (3)	C33—C34—C35	120.2 (3)
C15—C10—C9	120.9 (3)	C33—C34—H34	119.9
C11—C10—C9	119.7 (3)	C35—C34—H34	119.9
C12—C11—C10	120.1 (3)	C34—C35—C36	119.9 (3)
C12—C11—H11	120.0	C34—C35—H35	120.1
C10—C11—H11	120.0	C36—C35—H35	120.1
C13—C12—C11	120.0 (4)	C37—C36—C35	119.6 (3)
C13—C12—H12	120.0	C37—C36—H36	120.2
C11—C12—H12	120.0	C35—C36—H36	120.2
C12—C13—C14	120.4 (4)	C32—C37—C36	120.1 (4)
C12—C13—H13	119.8	C32—C37—H37	119.9
C14—C13—H13	119.8	C36—C37—H37	119.9
C13—C14—C15	120.5 (4)	N3—C38—C39	113.1 (3)
C13—C14—H14	119.8	N3—C38—H38A	109.0
C15—C14—H14	119.8	C39—C38—H38A	109.0
C14—C15—C10	119.8 (4)	N3—C38—H38B	109.0
C14—C15—H15	120.1	C39—C38—H38B	109.0
C10—C15—H15	120.1	H38A—C38—H38B	107.8
N1—C16—C17	110.5 (3)	C40—C39—C44	119.8 (4)
N1—C16—H16A	109.6	C40—C39—C38	119.4 (3)
C17—C16—H16A	109.6	C44—C39—C38	120.7 (3)
N1—C16—H16B	109.6	C39—C40—C41	119.6 (4)
C17—C16—H16B	109.6	C39—C40—H40	120.2
H16A—C16—H16B	108.1	C41—C40—H40	120.2
C18—C17—C22	119.9 (3)	C42—C41—C40	120.3 (4)
C18—C17—C16	120.3 (3)	C42—C41—H41	119.8
C22—C17—C16	119.7 (3)	C40—C41—H41	119.8
C17—C18—C19	120.3 (3)	C41—C42—C43	120.2 (4)
C17—C18—H18	119.8	C41—C42—H42	119.9
C19—C18—H18	119.8	C43—C42—H42	119.9
C20—C19—C18	119.9 (3)	C42—C43—C44	120.0 (4)
C20—C19—H19	120.0	C42—C43—H43	120.0
C18—C19—H19	120.0	C44—C43—H43	120.0
C21—C20—C19	119.6 (4)	C43—C44—C39	120.0 (4)
C21—C20—H20	120.2	C43—C44—H44	120.0
C19—C20—H20	120.2	C39—C44—H44	120.0
			
C6—C1—C2—C3	−0.2 (5)	C28—C23—C24—C25	0.3 (5)
C7—C1—C2—C3	−177.6 (3)	C29—C23—C24—C25	−179.0 (3)
C1—C2—C3—C4	−0.2 (6)	C23—C24—C25—C26	−1.8 (6)
C2—C3—C4—C5	0.5 (6)	C24—C25—C26—C27	2.8 (7)
C3—C4—C5—C6	−0.3 (6)	C25—C26—C27—C28	−2.3 (6)
C4—C5—C6—C1	−0.1 (6)	C24—C23—C28—C27	0.1 (5)
C2—C1—C6—C5	0.4 (5)	C29—C23—C28—C27	179.4 (3)
C7—C1—C6—C5	177.9 (3)	C26—C27—C28—C23	0.9 (6)
C8—N2—C7—O1	12.0 (5)	C30—N4—C29—O2	11.8 (5)
C8—N2—C7—C1	−168.0 (3)	C30—N4—C29—C23	−168.1 (3)
C2—C1—C7—O1	−178.0 (3)	C28—C23—C29—O2	174.3 (3)
C6—C1—C7—O1	4.6 (5)	C24—C23—C29—O2	−6.4 (5)
C2—C1—C7—N2	2.1 (5)	C28—C23—C29—N4	−5.8 (5)
C6—C1—C7—N2	−175.3 (3)	C24—C23—C29—N4	173.5 (3)
C16—N1—C8—N2	−19.0 (5)	C38—N3—C30—N4	174.2 (3)
C9—N1—C8—N2	177.2 (3)	C31—N3—C30—N4	−17.7 (5)
C16—N1—C8—S1	158.1 (3)	C38—N3—C30—S2	−7.3 (5)
C9—N1—C8—S1	−5.7 (5)	C31—N3—C30—S2	160.7 (3)
C7—N2—C8—N1	−61.4 (4)	C29—N4—C30—N3	−62.3 (4)
C7—N2—C8—S1	121.3 (3)	C29—N4—C30—S2	119.1 (3)
C8—N1—C9—C10	−146.6 (3)	C30—N3—C31—C32	−106.5 (4)
C16—N1—C9—C10	48.4 (4)	C38—N3—C31—C32	62.2 (4)
N1—C9—C10—C15	55.3 (5)	N3—C31—C32—C37	62.2 (4)
N1—C9—C10—C11	−128.9 (3)	N3—C31—C32—C33	−117.7 (3)
C15—C10—C11—C12	0.4 (5)	C37—C32—C33—C34	1.3 (5)
C9—C10—C11—C12	−175.5 (3)	C31—C32—C33—C34	−178.8 (3)
C10—C11—C12—C13	−0.2 (5)	C32—C33—C34—C35	−1.0 (5)
C11—C12—C13—C14	0.1 (6)	C33—C34—C35—C36	0.6 (6)
C12—C13—C14—C15	−0.2 (6)	C34—C35—C36—C37	−0.6 (6)
C13—C14—C15—C10	0.4 (6)	C33—C32—C37—C36	−1.3 (6)
C11—C10—C15—C14	−0.5 (5)	C31—C32—C37—C36	178.8 (4)
C9—C10—C15—C14	175.3 (3)	C35—C36—C37—C32	0.9 (6)
C8—N1—C16—C17	−103.7 (4)	C30—N3—C38—C39	−132.9 (3)
C9—N1—C16—C17	60.7 (4)	C31—N3—C38—C39	58.2 (4)
N1—C16—C17—C18	−123.1 (3)	N3—C38—C39—C40	−139.2 (3)
N1—C16—C17—C22	59.2 (4)	N3—C38—C39—C44	44.8 (5)
C22—C17—C18—C19	1.2 (5)	C44—C39—C40—C41	1.3 (5)
C16—C17—C18—C19	−176.5 (3)	C38—C39—C40—C41	−174.8 (3)
C17—C18—C19—C20	−1.0 (5)	C39—C40—C41—C42	0.5 (6)
C18—C19—C20—C21	0.2 (6)	C40—C41—C42—C43	−1.8 (6)
C19—C20—C21—C22	0.4 (6)	C41—C42—C43—C44	1.2 (6)
C20—C21—C22—C17	−0.2 (6)	C42—C43—C44—C39	0.7 (6)
C18—C17—C22—C21	−0.7 (5)	C40—C39—C44—C43	−1.9 (6)
C16—C17—C22—C21	177.1 (3)	C38—C39—C44—C43	174.1 (4)

### Hydrogen-bond geometry (Å, °)

**Table:**

*D*—H···*A*	*D*—H	H···*A*	*D*···*A*	*D*—H···*A*
N2—H2···S2	0.86	2.54	3.334 (3)	154
N4—H4···S1^i^	0.86	2.54	3.334 (3)	154
C13—H13···O2^ii^	0.95	2.57	3.193 (5)	124
C14—H14···O2^ii^	0.95	2.60	3.207 (5)	122
C25—H25···O1^iii^	0.95	2.55	3.228 (4)	129

Symmetry codes: (i) *x*−1, *y*, *z*; (ii) −*x*+3, *y*+1/2, −*z*; (iii) −*x*+2, *y*−1/2, −*z*.
